# Flavonoid Rutin Presented Anti-Glioblastoma Activity Related to the Modulation of Onco miRNA-125b Expression and STAT3 Signaling and Impact on Microglia Inflammatory Profile

**DOI:** 10.3390/brainsci14010090

**Published:** 2024-01-17

**Authors:** Irlã Santos Lima, Érica Novaes Soares, Carolina Kymie Vasques Nonaka, Bruno Solano de Freitas Souza, Balbino Lino dos Santos, Silvia Lima Costa

**Affiliations:** 1 Laboratory of Neurochemistry and Cellular Biology, Institute of Health Sciences, Federal University of Bahia, Salvador 40231-300, Brazil; irlalima@ufba.br (I.S.L.); ericanovaessoares@gmail.com (É.N.S.); 2 Center of Biotechnology and Cell Therapy, São Rafael Hospital, D’Or Institute for Research and Teaching (IDOR), Salvador 41253-190, Brazil; carolina.nonaka@hsr.com.br (C.K.V.N.); brunosolanosouza@gmail.com (B.S.d.F.S.); 3 College of Nursing, Federal University of Vale do São Francisco, Petrolina 56304-917, Brazil; 4 National Institute of Translation Neuroscience (INNT), Rio de Janeiro 21941-902, Brazil

**Keywords:** glioblastoma, miRNA-125b, STAT3, inflammatory cytokines, rutin

## Abstract

Glioblastoma (GBM) is the most aggressive and treatment-resistant brain tumor. In the GBM microenvironment, interaction with microglia is associated with the dysregulation of cytokines, chemokines, and miRNAs, contributing to angiogenesis, proliferation, anti-apoptosis, and chemoresistance. The flavonoid rutin can inhibit glioma cell growth associated with microglial activation and production of pro-inflammatory mediators by mechanisms that are still poorly understood. The present study investigated the effect of rutin on viability, regulation of miRNA-125b, and the STAT3 expression in GBM cells, as well as the effects on the modulation of the inflammatory profile and STAT3 expression in microglia during indirect interaction with GBM cells. Human GL15-GBM cells and human C20 microglia were treated or not with rutin for 24 h. Rutin (30–50 μM) significantly reduced the viability of GL15 cells; however, it did not affect the viability of microglia. Rutin (30 μM) significantly reduced the expression of miRNA-125b in the cells and secretome and STAT3 expression. Microglia submitted to the conditioned medium from GBM cells treated with rutin showed reactive morphology associated with reduced expression of IL-6, TNF, and STAT3. These results reiterate the anti-glioma effects of the flavonoid, which may also modulate microglia towards a more responsive anti-tumor phenotype, constituting a promising molecule for adjuvant therapy to GBM.

## 1. Introduction

Glioblastoma (GBM) is a highly aggressive brain tumor whose complete surgical resection is challenging due to its infiltrative nature [1]. Standard therapy involves surgery for tumor resection, followed by radiotherapy and chemotherapy, but the median survival is limited to about 15 months [2,3]. The tumor microenvironment (TME) emerges as a crucial factor in GBM progression, involving complex interactions between tumor cells and mesenchymal cells, glial cells, stem cells, fibroblasts, vascular cells, and tumor-associated macrophages (TAM) [3]. The activation of microglia, which is essential for the development of the central nervous system (CNS), plays an ambivalent role and may promote tumorigenesis or inflammatory response in the GBM [4]. This process acts as a vicious cycle, in which M2-type TAM cells are stimulated by the tumor itself, releasing factors like TNF and interleukins such as IL-6, IL-1b, and IL-10, which promote tumor proliferation and survival. An alternative to interrupting this cycle can be the inhibition of the anti-inflammatory phenotype of TAMs and far repolarization towards an inflammatory profile [5,6,7]. On the other hand, the activation of signaling pathways such as NFκB by TNF by microglia, astrocytes, or glioma cells themselves can induce an increase in IL6 expression, which can activate the JAK/STAT3 pathway and contribute to tumor proliferation, migration, and invasion. All these factors are associated with a poor prognosis [8,9,10]. Furthermore, several molecules that have epigenetic capacity have an impact on the regulation of TME plasticity. Several epigenetic modifications have been associated with the biological characteristics of this tumor, some playing essential roles as therapeutic targets [11]. In this context, there is evidence that miRNAs, which are small RNAs, do not have a protein-coding function. Nevertheless, they bind to mRNAs and play crucial roles in gene regulation [12,13]; miRNAs, such as miR125b, emerge as crucial components in oncogenic upregulation and are associated with the STAT3 signaling pathway [14,15]. Studies have pointed out that the modulation of miRNA expression by tumor cells associated with proliferation suppression can increase drug sensitivity and suppress metastasis and angiogenesis. Strategies to disrupt this mechanism include inhibition of miRNA-125b and repolarization of TAMs towards an inflammatory profile.

The flavonoid rutin, a glycone of quercetin, is widely distributed in plants [16] and has been associated with several beneficial pharmacological properties, including anti-inflammatory, neuroprotective, antiproliferative, anticarcinogenic, stress antioxidant, and anticancer effects [17]. According to transcriptome studies developed by bioinformatics tools, rutin can participate in the regulation of miRNAs [18]. In vitro and in vivo studies have demonstrated the impact of this natural agent on the regulation of different molecular mechanisms, such as Wnt/β-catenin, p53-independent pathway, PI3K/Akt, MAPK, p53, apoptosis and NF-ĸB, and JAK /STAT, which help mediate its anti-cancer impacts [19]. Furthermore, it was demonstrated that, combined with TMZ treatment, rutin increased the cytotoxicity and inhibition of cytoprotective autophagy of GBM cells [20]. Rutin also significantly reduced the expression of inflammatory mediators such as IL-6, TNF-α, IL-1β, and NO in microglial cells from BV-2 rats after stimulation with LPS [21]. In studies developed by our group, the properties of rutin were initially characterized at concentrations of 1 to 100 μM, which induced cytotoxicity and inhibited the proliferation of human GBM cells associated with the modulation of the ERK/MAPK signaling pathway [22]. Rutin was also able to inhibit GBM cell migration associated with the reduction of expression of extracellular components and matrix-associated metalloproteinases [22]. Subsequently, we demonstrated that rutin can modulate the inflammatory profile of isolated rat microglia [23] and, more recently, we have shown that this flavonoid and its aglycone quercetin exhibit anti-glioma effects associated with the property of modulating the inflammatory profile of microglia. In the study developed by Amorim et al. (2020) [24], it was also demonstrated that the rutin flavonoid can reduce the proliferation of tumor cells, as well as induce the chemotaxis of microglia to the tumor microenvironment in monocultures of cells of the C6 lineage, stimulate the upregulation of tumor necrosis factor (TNF) expression, and reduce the expression of cytokines and chemokines such as IL-10, MCI, and growth factors (IGF, GDNF). The antitumor effect of this molecule can also be observed in an indirect coculture model (via glioma conditioning medium), inducing microglial regulation to a pro-inflammatory profile by increasing the expression levels of cytokines such as IL-1β, IL-6, and IL-18.

In this context, in the present study, we analyzed the anti-glioma effects of rutin on viability, miRNA-125b expression, and STAT3 expression in human GBM cells, as well as its immunomodulatory property during indirect interaction (via secretome) with human microglia, relating inflammatory mediators and modulating STAT3 signaling. The results herein presented reiterate the anti-glioma potential of the flavonoid and reveal its property in modulating the expression of the onco miRNA-125b, which may be implicated in the modulation of the inflammatory profile of microglia towards a more responsive antitumor phenotype. Therefore, this work can contribute to a better understanding of miRNAs, target mechanisms, and immunological response associated with rutin treatment, offering valuable insights to guide more effective strategies, consolidating the basis for the successful application of rutin in adjuvant therapies in the treatment of GBM.

## 2. Results

### 2.1. Rutin Selectively Reduces the Viability of hGBM Cells without Affecting Microglial Viability

**Figure 1:**
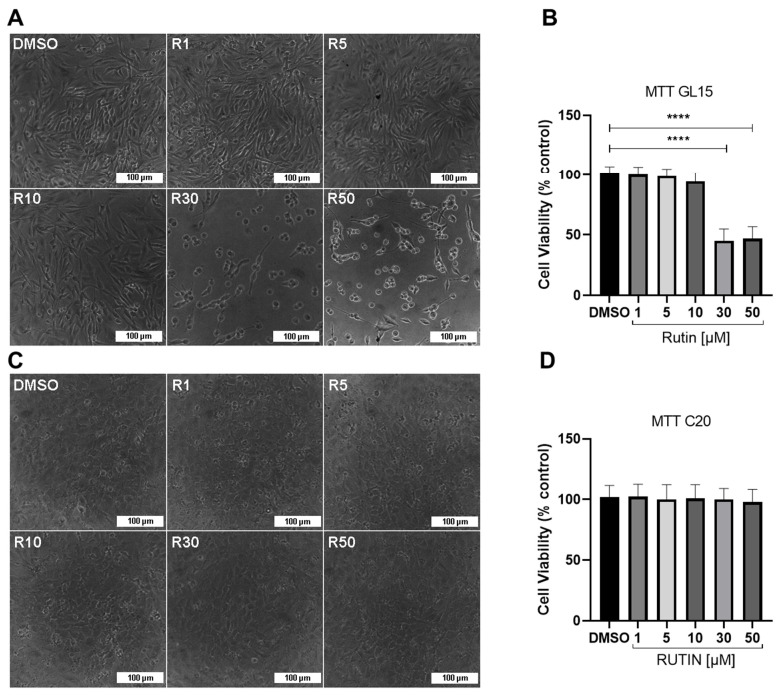
Effect of rutin on the viability of GL15 human glioblastoma cells and C20 human microglia. The cells were treated for 24 h with different concentrations of rutin (1, 5, 10, 30, and 50 μM) or maintained under control conditions (0.05% DMSO). (**A**,**C**) Phase contrast photomicrographs of GL15 and C20 cell cultures in different treatments; scale bar = 100 μm. (**B**,**D**) Analysis by MTT test of cell viability in GL15 and C20 cells in different treatments; values were expressed as the means ± SD (*n* = 3); the results were compared to controls (100%), and the significance was evaluated by a one-way ANOVA test followed by the Tukey test; **** *p* < 0.0001.

To analyze the effects of the flavonoid rutin in GL15 cell viability, which is derived from human GBM, and C20 cell viability, which is an immortalized human microglia cell, we conducted a study at different concentrations of the flavonoid (1–50 μM). The cell viability was determined by MTT, and the morphology of the cells well was analyzed by interference microscopy with phase contrast (Figure 1A–D). We observed that 24 h after the treatments, GBM cells treated with rutin at concentrations of 1, 5, and 10 μM presented morphology similar to the control. However, in the cultures treated with 30 and 50 μM rutin, there was a significant reduction in cell density and remaining adherent cells showed rounded morphology with contracted cytoplasm (Figure 1A), and there was a significant decrease (>50%) in cell viability in the treated cultures compared to the control (Figure 1B). On the other hand, no significant difference was observed in the morphology and viability in the cultures of C20 cells treated with rutin (Figure 1C,D). 

### 2.2. Rutin Regulates the Expression of miRNAs-125b in GBM Cells

**Figure 2:**
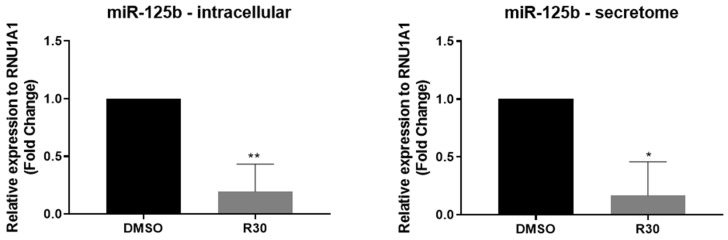
Effect of the flavonoid rutin on the regulation of miRNA-125b in GL15 human GBM cells. miRNA analyses using RT-qPCR. GL15 cells were treated for 24 h with rutin at 30 µM (R30) or maintained under control conditions (0.03% DMSO). The expression of intracellular miRNA-125b and the extracellular matrix (secretome) were analyzed. Values were expressed as means ± SD (*n* = 3); results expressed are relative to control and treatment; significance was determined by an unpaired *t*-test; ** *p* < 0.002; * *p* < 0.02.

We investigated the expression of miRNAs in GL15 cells at the intracellular level and in the extracellular matrix (secretome). Based on dose-dependent effects on the viability of GL15 GBM cells, the cells were treated with rutin at 30 μM or kept under control conditions (0.03% DMSO) and the expression of the onco miRNA-125b was analyzed after 24 h in the cells and in the secretome using RT-qPCR. We observed that GBM cells express and secrete miRNA-125b, and the treatment with rutin induces a highly significant reduction in the levels of this miRNA at both intracellular (** *p* < 0.002) and secretome (* *p* < 0.02), compared with control cultures (Figure 2).

### 2.3. Rutin Modulates the Expression of STAT3 in GBM Cells

**Figure 3:**
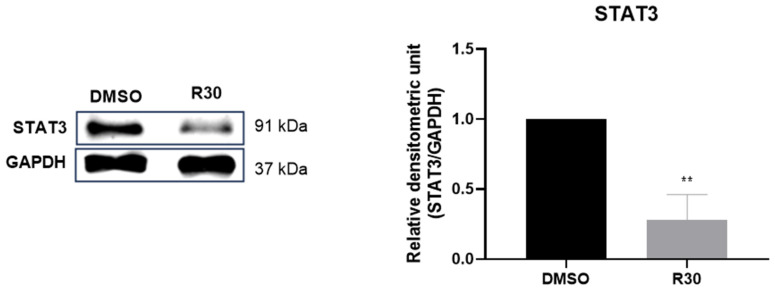
Effect of rutin on the regulation of STAT3 protein expression in GBM cells. GL15 cells were subjected to rutin treatment at a concentration of 30 µM (R30) or maintained under control conditions (0.03% DMSO), and STAT3 protein expression was evaluated by Western blot after 24 h. The results were normalized to the intensity of the reference protein, GAPDH, and significance was determined by an unpaired *t*-test; values were expressed as means ± SD (*n* = 3); ** *p* < 0.002.

To evaluate whether the STAT3 inflammatory signaling pathway is involved in the rutin effects on GBM cells, we analyzed the expression levels of STAT3 in GL15 cells treated with the flavonoid (30 µM) or maintained under control conditions (0.03% DMSO) after 24 h by Western blot technique. The data obtained indicated that the exposure of GL15 cells to rutin induced a significant (** *p* < 0.002) negative regulation in STAT3 protein expression (Figure 3).

### 2.4. The Treatment of GBM Cells with the Flavonoid Rutin Induces a Change in the Morphology of Microglia

**Figure 4:**
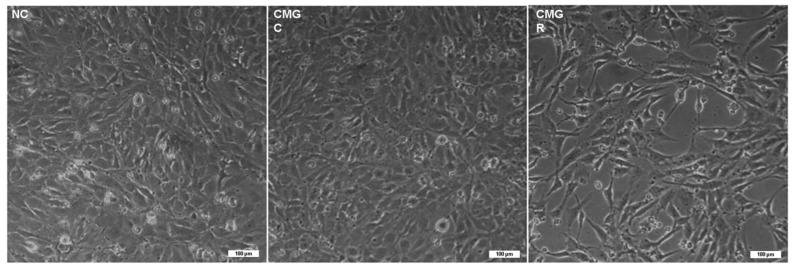
Effect of conditioned medium derived from GL15 GBM cells treated with the flavonoid rutin on the morphology of microglia C20. GL15 cells were treated for 24 h with rutin (30 µM) or maintained under control conditions (0.03% DMSO). C20 microglia were exposed to fresh medium including culture with fresh medium as a negative control (NC), to the culture medium from GL15 cells under control conditions treated with 0.03% DMSO (CMGC), or to the culture medium of GL15 cells treated with the flavonoid rutin at 30 µM (CMGR) for 24 h. The results represent three independent experiments. Phase-contrast photomicrographs of GL15 and C20 cells illustrate morphological differences between treatment and control; scale bar = 100 µm.

To better understand the characterization of the microglial response to the exposure to the secretome of GBM cells, indirect interaction assays were performed. In these assays, cultures of human C20 microglia were exposed for 24 h to fresh medium as a negative control (NC), conditioned medium (CM) generated by GL15 cells under control conditions (CMGC), or conditioned medium generated after treatment with rutin at a concentration of 30 µM (CMGR). With phase contrast microscopy it was possible to analyze the morphology of C20 cells in different conditions, revealing a significant difference between the NC-, CMGC-, or CMGR-treated groups after 24 h (Figure 4). Only C20 microglia exposed to CMGR exhibited an elongated cell body and increased cellular processes, and the cellular layer showed some gaps juxtaposed with cells presenting this phenotype, suggesting a reactive response. However, this morphological pattern was not observed in C20 cells directly treated with rutin at the concentrations tested (1, 5, 10, 30, and 50 µM) (Figure 1C).

### 2.5. The Treatment of GBM Cells with Rutin Indirectly Regulates the Expression of Inflammatory Mediators in Microglia

**Figure 5:**
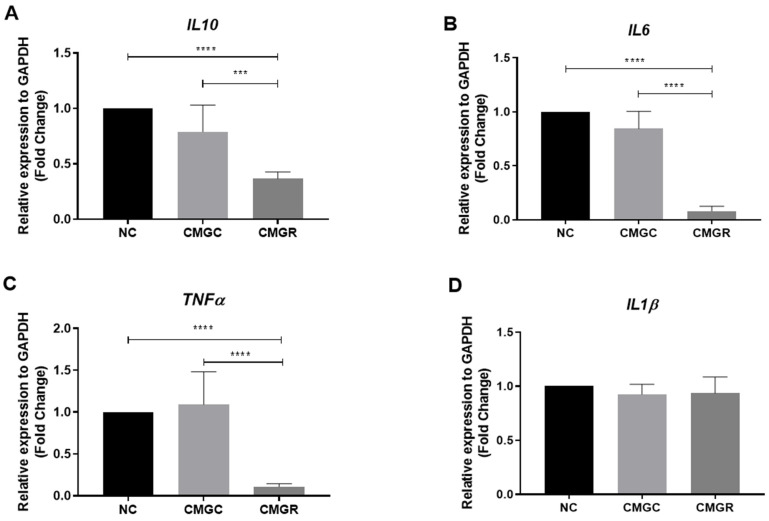
Effect of conditioned medium derived from glioblastoma GL15 cells treated with the flavonoid rutin on the expression of mRNA for cytokines IL10 (**A**), IL6 (**B**), TNFα (**C**) and IL1β (**D**) by C20 microglia. GL15 cells were treated for 24 h with rutin at a concentration of 30 µM (R30) or maintained under control conditions with (0.03% DMSO). C20 microglia were exposed to fresh medium as a negative control (NC), to the culture medium from GL15 cells under control conditions (treated with 0.03% DMSO, CMGC), or to the culture medium from GL15 cells treated with the flavonoid (CMGR). The cytokine expression was analyzed by RT-qPCR after 24 h. Values were expressed as means ± SD (*n* = 3). The significance was evaluated by a one-way ANOVA test followed by the Tukey test; **** *p* < 0.0001; *** *p* < 0.0002.

We investigated the expression of inflammatory mediators (IL-6, IL-10, IL-1β, and TNF-α) by RT-qPCR in microglial cells (C20) in GL15 cells cultured for 24 h under negative control conditions, which was fresh medium without FBS(NC), treated with conditioned medium containing the secretome of GL15 cells under control conditions (CMGC) or treated with rutin at 30 µM (CMGR) for 24 h (Figure 5). We observed a significant reduction in the levels of the regulatory cytokine IL-10 in cultures treated with CMGR compared to the NC group and to the control conditioned medium (CMGC); however, there was no significant difference in cultures of C20 treated with control conditioned medium (CMGC) (**** *p* < 0.0001 and *** *p* < 0.0002, respectively) (Figure 5A). Under the same experimental conditions, we analyzed the expression of the inflammatory cytokines IL-6 and TNF-α. Remarkably, there was a significant reduction (**** *p* < 0.0001) in the expression of these cytokines when cells were exposed to CMGR compared to both CN and CMGC controls (Figure 5B,C). On the other hand, no statistically significant differences were observed in the expression of IL-1β under any of the treatment conditions (Figure 5D).

### 2.6. The Treatment of GBM Cells with the Flavonoid Rutin Negatively Regulates the Pro-Tumorigenic Signaling Pathway STAT3 in Microglia

**Figure 6:**
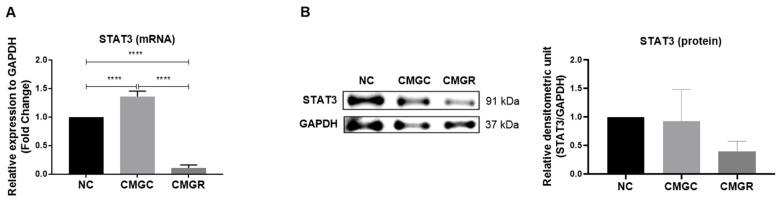
Effect of conditioned medium derived from glioblastoma GL15 cells treated with the flavonoid rutin on the mRNA and protein expression of STAT3. Assessments were made using RT-qPCR and Western blot techniques. C20 microglia were exposed to fresh medium as a negative control (NC), to the culture medium of GL15 cells under control conditions treated with 0.03% DMSO (CMGC), or to the culture medium of GL15 cells treated with the flavonoid rutin at 30 µM (CMGR) for 24 h. (**A**) STAT3 mRNA expression in microglia cells by RT-qPCR; (**B**) immunoreactive bands of STAT3 and GAPDH proteins in microglia and relative expression of STAT3 in microglia. Values were expressed as means ± SD (*n* = 3). The results were normalized to the intensity of the reference protein GAPDH. Significance was determined by a one-way ANOVA test followed by the Tukey test; **** *p* < 0.0001.

We investigated the expression of STAT3 in C20 microglia using RT-qPCR and Western blot techniques with different experimental conditions, including culture with fresh medium as negative control (NC), treatment with conditioned medium containing the secretome of GL15 cells under control conditions using 0.03% DMSO (CMGC), and treatment with conditioned medium containing secretome from GL15 cells previously treated with 30 µM rutin (CMGR) (Figure 6). We observed that 24 h exposure of microglia to CMGC induced a significant increase (**** *p* < 0.0001) in STAT3 mRNA expression compared to NC. On the other hand, exposure to CMGR resulted in a significant reduction (**** *p* < 0.0001) in STAT3 mRNA expression in C20 cells (Figure 6A). Significant changes in STAT3 protein expression were also observed in the different conditions evaluated (Figure 6B). Therefore, treatment of hGBM cells with rutin for 24 h was able to induce a significant reduction (**** *p* < 0.0001) in the expression levels of both STAT3 mRNA and protein in microglia.

## 3. Discussion

The results obtained in this study are consistent with previous research using rutin, observing a significant reduction after 24 h treatment in the viability of rat C6 glioma cells and human GBM cells (GL15, U251, and TG1) at concentrations near or above 50 µM [22,25,26]. In this study, we conducted experiments with concentrations ranging from 1 to 50 µM and observed that the flavonoid at a concentration of 30 µM was sufficient to reduce the viability of human GL15 cells by around 50% within 24 h, without affecting the viability of C20 microglia cells.

Our research also aimed at contributing to the understanding of the complex interactions between GBM cells and other cells from the TME, providing valuable insights for future therapeutic approaches and research in brain cancer. Hence, we investigated the expression of miRNA-125b, considering that an in vitro study demonstrated that its positive expression stimulates the proliferation of human GBM cells while inhibiting apoptosis induced via Bcl-2 regulation [27]. Additionally, Smits et al. (2012) [28] showed that miRNA-125b expression induces angiogenesis, and Shi (2011) [29] observed its association with resistance to temozolomide in GBM treatment. Based on our results, we found that rutin reduced the expression levels of miRNA-125b in hGBM cells. This study represents, to our knowledge, the first evidence of the impact of rutin on the negative regulation of onco miRNAs. Signaling pathways play a crucial role in GBM biology, including the STAT3 and NFκB pathways. There is a significant interconnection between these pathways, resulting in complex crosstalk. This interaction may have a regulatory impact on pro-tumorigenic molecules [8,30]. In this context, as demonstrated by Parisi et al. (2016) [31], miRNA-125b is implicated in the regulation of the STAT3 signaling pathway and in the activation of microglia. Therefore, we analyzed the expression levels of STAT3 protein in GBM cells. A significant reduction in STAT3 protein was observed in the GL15 cells treated with the flavonoid rutin compared to the control. The reduction of STAT3 expression associated with the reduction of miRNA-125b suggests that rutin may influence the STAT3 and signaling pathways regulated by this miRNA.

Moreover, our investigation aimed to clarify whether rutin can modulate the microglia inflammatory profile during interaction with GBM cells and could have an impact on tumor sensibilization. As observed in previous studies, rutin has the potential to modulate the inflammatory profile of rat microglial cells in vitro, leading to significant changes after 24 h of treatment [23]. Based on the research conducted by da Silva et al. (2020) [25], which highlighted the ability of the flavonoid to modulate the inflammatory profile of microglia during interaction with rat glioma C6 cells, either through direct co-cultures or indirect interactions (via microglia secretome or C6 cells treated with the flavonoid), our current study aimed at gaining a deeper understanding of the microglial response to exposure to the secretome of human GBM cells (GL15). It became evident that when C20 microglia are treated with CMGR, changes in morphology occur, indicating possible glial reactivity. We also investigated the effects of this indirect interaction in the expression of cytokines IL-6, IL-10, IL-1β, and TNF-α, and in the STAT3 signaling protein in microglia subjected to a conditioned medium containing secretome from GBM cells treated or untreated with rutin, as well as under more homeostatic control conditions. We observed that the treatment of microglia with conditioned medium containing either control (CMGC) or rutin-treated (CMGR) secretome did not influence the mRNA expression of the cytokine IL-1β. IL-1β plays a relevant role in the activation of various signaling pathways, including the NFκB transcription factor, which regulates the production and release of pro-inflammatory mediators essential for the development and progression of glioma [32]. The lack of significant changes in IL-1β expression may indicate a highly controlled regulation or the influence of other factors on its expression. Furthermore, the modulation of IL-1β may depend on different regulators and cellular contexts, including other signaling pathways [33]. As revised by Nascimento et al. (2021) [34], in the GBM TME, IL-10 is positively regulated, and microglia shifts towards M2-like characteristics, contributing to the production of inflammatory cytokines. Through analysis, we observed a significant reduction in IL-10 mRNA levels in the CMGR-treated group compared to the NC group. However, no significance was found in the expression of IL-10 in microglia treated exclusively with CMGR. These results suggest that CMGR, composed of the secretome of GL15 cells after rutin treatment, may have the capacity to modulate IL-10 expression in microglia in a specific context, possibly mediated by complex interactions between secretome components and microglial cells. However, it is important to note that the lack of statistical significance in IL-10 expression in microglia treated exclusively with CMGR suggests that this influence may depend on additional factors or specific cellular interactions. On the other hand, we observed that CMGR can induce a significant reduction in mRNA expression for the cytokines IL-6 and TNF in microglia. These cytokines are associated with inflammatory regulation in the TME [30,35], and they may have important implications for modulating the immune and inflammatory response in the GBM environment, especially in immunomodulating the microglial profile. Our findings are in line with the results of Silva et al. (2020) [25], who demonstrated a reduction in the expression of IL-6 and IL-10 levels in rat microglia cultures treated with C6 glioma cell secretome exposed to rutin at 50 µM for 24 h. In contrast, an increase in IL-1β and TNF levels was observed in these same results. Differences in cell lines and the doses of rutin used may explain the differences in responses observed. These complexities highlight the importance of interpreting results, taking into account the specific contexts of each study.

STAT3 is highly activated in the TME, and besides its high expression in GBM cells, it is also associated with microglial modulation in this environment [36]. In the indirect interaction between GBM under control conditions (CMGC), we observed positive regulation in STAT3 mRNA expression in microglia. On the other hand, microglia exposed to CMGR showed a significant reduction in STAT3 mRNA and protein expression compared to microglia in the NC group. Considering the role of the signaling protein STAT3 in the expression of inflammatory cytokines [37], the reduction of its expression in CMGR-treated microglia may also be implicated in the negative regulation of hGBM cells’ miRNA-125b, as well as IL-6 mRNA, which may be related to reductions in TNF mRNA expression in the context of change in the inflammatory profile. This observation highlights the complexity of the STAT3 pathway and the need to consider multiple aspects of the regulation of signaling pathways, such as the NFκB pathway, which is actively associated with inflammatory mediators in the GBM TME.

Although this evidence suggests the positive impact of rutin on anti-glioma actions, it is essential to conduct a more comprehensive and in-depth analysis of the dysregulation of specific molecules and the intricate mechanisms associated with GBM progression. The intrinsic heterogeneity of GBM, evidenced by the molecular diversity between different tumor lineages, justifies the need to include other GBM lineages in these investigations. This approach allows us to cover the different gene expressions, molecular profiles, and cellular responses, which are essential for a more complete understanding of the impact of rutin. Furthermore, the inclusion of analyses on explants from glioblastoma patients is also crucial, enabling the validation and contextualization of results in a scenario closer to real clinical conditions. Such diverse approaches would strengthen the scientific basis, enriching conclusions and contributing to a more comprehensive and translational approach in developing therapeutic strategies for GBM. The results of the present study, together with previous studies by us and others, consolidate the scientific basis for the use of rutin as an adjuvant in the treatment of GBM, which may be considered in other translational and clinical studies. 

## 4. Materials and Methods

### 4.1. Cell Culture

The GL15 cell line (passages 120–130) established from a human GBM by Bocchini et al. (1991) [22,38] was chosen for its proliferation, migration, invasion, and resistance properties, and it was cultured in Dulbecco’s Modified Eagle Medium (DMEM: Island Biological Company-GIBCO ^®^, Grand Island, NY, USA), containing 7 mmol/L glucose, 2 mmol/L L-glutamine, and 0.011 g/L pyruvic acid, as previously described by Santos et al. 2015 [22]. The immortalized primary human microglia C20 cell line, originally developed and characterized by Garcia-Mesa et al. (2017) [39] and kindly provided by Dr. Henning Ulrich from the Department of Biochemistry, Institute of Chemistry at the University of São Paulo (USP), was cultured in DMEM F12 50/50 medium as described by the authors [39]. Both cultures were supplemented with 10% fetal bovine serum (FBS) and antibiotics (100 U/mL penicillin and 100 µg/mL streptomycin, Gibco ^®^) and maintained in an incubator under standardized conditions of a humidified atmosphere with 5% CO_2_ at a temperature of 37 °C. Cells were cultured in 100 mm polystyrene plates (TPP, Trasadingen, Switzerland), following the protocol described by Santos et al. (2015), until reaching the desired confluence. Upon reaching confluence, the medium was removed, and adherent cells were detached using a trypsin solution (0.05% trypsin and 0.02% EDTA in PBS) and seeded into 6- or 96-well polystyrene plates (Kasvi, São José dos Pinhais, SP, Brazil), according to the experiment, at a density of 5 × 10^4^ cells/cm^2^. 

### 4.2. Treatment Drugs

Rutin (3-rutinoside of 3,3′,4′,5,6-pentahydroxyflavone) was obtained from Merck (Boston, MA, USA) (R5143) and dissolved in dimethyl sulfoxide (DMSO; Sigma, Tokyo, Japan) to form a 100 mM stock solution, which was stored and protected from light at −4 °C. At the time of treatments, GL15 cells were incubated for 24 h with rutin at concentrations varying between 1, 5, 10, 30, and 50 µM, depending on the experiment, in an attempt to investigate the most appropriate dose response. The vehicle for diluting flavonoids, dimethyl sulfoxide (DMSO), used to demonstrate cultivation under control conditions in a volume equivalent to the maximum concentration adopted in flavonoids (0.05%), was diluted directly in culture medium without fetal bovine serum (FBS) and did not show a significant effect on the parameters analyzed when compared to cultures that were not exposed to this solvent.

### 4.3. Cell Viability

To evaluate the viability of human glioblastoma GL15 and human microglia C20 cell lines, they were seeded in a 96-well plate (Kasvi) with an approximate cell density of 2.2 × 10^4^ cells/cm^2^, corresponding to approximately 8000 cells per well, and cultured in fresh medium DMEM or DMEM F12 properly supplemented with SFB. Thus, they were incubated for 24 h in standardized conditions of a humidified atmosphere with 5% CO_2_ at a temperature of 37 °C. Cell viability was determined by the conversion of the yellow salt 3-(4,5-dimethylthiazol-2-yl)-2,5-diphenyl tetrazolium bromide (MTT) into formazan crystals (purple) by mitochondrial dehydrogenases of live cells. After 24 h of plating, cells were treated with the previously defined concentrations. After 24 h of treatment, cells were incubated with an MTT solution (Thermo Fisher, Waltham, MA, USA, 0.5 mg MTT per 1 mL) at 37 °C and 5% (*v*/*v*) CO_2_ for 2 h. Subsequently, 100 µL of a lysis buffer containing 20% (*w*/*v*) sodium dodecyl sulfate (SDS), 50% (*v*/*v*) acetic acid, and 2.5% (*v*/*v*) 1 mol/L HCl were added, and the plates were incubated for 6 h. The optical density of the samples was measured using a spectrophotometer (Varioskan™ Flash Multimode Reader, Thermo Plate) at a wavelength of 540 nm. Three independent experiments with eight replicates for each variable were conducted, and the results were expressed as the percentage of viability of the treated groups relative to the control, which was considered 100%.

### 4.4. Culture with Indirect Interaction between GL15 and C20 Cells

For studies involving indirect interactions, cells were cultured in 6-well plates (Kasvi) at a density of 5 × 10^4^ cells/cm^2^. The GBM GL15 cells were cultured in fresh medium DMEM appropriately supplemented with FBS. Thus, they were incubated for 24 h under standardized conditions of a humidified atmosphere with 5% CO_2_ at a temperature of 37 °C. After 24 h treating GL15 GBM cells under control conditions (0.03% DMSO) or with rutin (30 µM), the conditioned media (CM) of the cultures, containing the secretome produced by GL15 cells, were collected and centrifuged at 2000× *g* for 5 min to remove any cellular debris. The CM was immediately used to treat human microglia C20 cells (indirect interaction) at a 1:4 ratio (fresh medium:CM). GL15 cells were collected and prepared for miRNA extraction following the manufacturer’s protocol using the miRNeasy kit (Qiagen, Hilden, Germany). After 24 h treating C20 cells with CM from GL15 cells treated with rutin (Rutin-treated GL15 conditioned medium—CMGR) or under control conditions (Control GL15 conditioned medium—CMGC), cells were collected for RNA extraction using Trizol® reagent (Invitrogen, Waltham, MA, USA, Life Technologies, Carlsbad, CA, USA, 15596026), following the manufacturer’s protocol. The experiments were performed in triplicate.

### 4.5. Analysis of miRNA Expression by RT-qPCR

The pellet samples of GL15 human GBM cells containing approximately 1 × 10^6^ cells were mixed with 700 μL of QIAzol Lysis Reagent from the miRNeasy kit (Qiagen). For the isolation of miRNAs from the cell culture supernatant, the miRNeasy Serum/Plasm Advanced kit (Qiagen) was used. For the supernatant, 5 times the volume of QIAzol Lysis Reagent provided by the manufacturer was added. The samples were vortexed for 1 min. Chloroform was added in the recommended volume for each kit, vigorously mixed for 15 s, and incubated for 3 min at room temperature. Subsequently, the samples were centrifuged for 15 min at 12,000× *g* at 4 °C. After centrifugation, the aqueous phase was collected and transferred to a new 1.5 mL tube (approximately 350 μL). Next, 1.5 times the volume (525 μL) of 100% ethanol was added and homogenized using a pipette for each sample. The samples were then transferred to a column (RNeasy MinElute spin column) provided by the manufacturer and centrifuged for 15 s at ≥10,000× *g* at room temperature. The liquid passing through the column of each sample was discarded, and the column was washed with 700 μL of Buffer RWT and centrifuged for another 15 s at ≥10,000× *g* at room temperature. Again, the liquid passing through the column of each sample was discarded, and the column was washed with 500 μL of Buffer RPE and centrifuged for 15 s at ≥10,000× *g* at room temperature. Then, the column of each sample was washed with 500 μL of 80% ethanol and centrifuged for 2 min at ≥10,000× *g* at room temperature. The columns were transferred to new properly labeled 1.5 mL tubes and left with the cap open for 5 min to evaporate residual ethanol. Thirty microliters of RNase-free ultrapure water provided by the manufacturer were added, followed by centrifugation for 1 min at maximum speed. The samples were stored at −80 °C until the next step. The experiments were conducted in triplicate. For the extraction of miRNAs from the cell culture supernatant, the miRNeasy Serum/Plasma kit (Qiagen) was used following the manufacturer’s recommendations. For cDNA synthesis, the miScript II RT Kit (Qiagen) was used with 10 ng of RNA quantified by Nanodrop™ 2000 spectrophotometer (Thermo Fisher Scientific), according to the manufacturer’s recommendations. The samples were incubated for 60 min at 37 °C, 95 °C for 5 min, and immediately placed on ice. Five microliters of diluted cDNA (1:20), 5 µL of SYBR™ Green PCR Master Mix (Thermo Fisher Scientific), and 1 µL of the commercial primer set miRCURY LNA (Qiagen) were used for a final volume of 10 µL. The amplification was performed on an ABI7500 FAST thermocycler (Applied Biosystem, Waltham, MA, USA). The endogenous control RNU1A1 was used for result normalization. The expression of miRNA levels was calculated using the 2^−ΔΔCT^ method [40] and analyzed using GraphPad Prism v 9.1.1 2020 (La Jolla, CA, USA).

### 4.6. Analysis of mRNA Expression by RT-qPCR

To analyze the expression of inflammatory cytokines by C20 microglia under control conditions (fresh medium), or treated with the conditioned medium from GL15 cells cultured for 24 h under control conditions (CMGC), or treated with the conditioned medium from GL15 cells cultured for 24 h in the presence of the flavonoid rutin at 30 μM (CMGR), cells were cultured in 6-well plates (Kasvi) with a cell density of approximately 1 × 10^6^ cells/cm^2^ and incubated for 24 h under standardized conditions of a humidified atmosphere with 5% CO_2_ at a temperature of 37 °C. After 24 h of treatments, the total RNA was extracted using Trizol^®^ reagent (Thermo Fisher Scientific) following the recommended manufacturer’s protocol. The experiment was performed in biological triplicate. RNA quantification was carried out using NanoDrop™ 2000 (Thermo Fisher Scientific). The samples were stored at −80 °C until further use. For the cDNA reaction, 1.5 µg of RNA and the commercial High-Capacity cDNA Reverse Transcription kit were used, following the manufacturer’s recommendations (Thermo Fisher Scientific). The cDNA was stored at −20 °C until use. Subsequently, real-time quantitative PCR (RT-qPCR) was performed on the ABI7500 FAST instrument (Applied Biosystems) under standard Taqman thermal cycling conditions by the manufacturer. The expressions of mRNAs in treated samples and control conditions were evaluated using commercial TaqMan^®^ probes: IL-6 (Hs00174131_m1), IL-10 (Hs00961622_m1), TNF-α (Hs00174128_m1), and IL-1β (Hs01555410_m1). The reference gene GAPDH (Hs99999905_m1) (Thermo Fisher Scientific) was used as a normalizer. The cDNA samples were diluted 1:100, 5 µL of TaqMan Universal Master Mix (Thermo Fisher Scientific) and 0.5 µL of specific TaqMan^®^ probes for each monoplex reaction were added to achieve a final volume of 10 µL. Expression analyses of STAT3 were performed by RT-qPCR assays using SYBR™ Green PCR Master Mix and the following primers: STAT3 Forward (5′ to 3′): ACCAGCAGTATAGCCGCTTC, STAT3 Reverse (5′ to 3′): GCCACAATCCGGGCAATCT, and the endogenous control GAPDH Forward (5′ to 3′): GCCAGCATCGCCCCACTTG, GAPDH Reverse (5′ to 3′): GTGAAGGTCAACGGAT. The expression levels of mRNAs were calculated using the 2^−ΔΔCT^ method (Schmittgen and Livak, 2008) [40] and analyzed using GraphPad Prism v 9.1.1 (2020).

### 4.7. Analysis of Signaling Pathways by Western Blot

The analysis of the effect of rutin on the expression of proteins involved in cellular signaling was conducted on human GL15 GBM cells treated directly with the flavonoid (30 μM), as well as on human C20 microglia cells under control conditions (fresh medium), or treated with the conditioned medium from GL15 cells cultured for 24 h under control conditions (CMGC), or treated with the conditioned medium from GL15 cells cultured for 24 h in the presence of rutin at 30 μM (CMGR). Cells were cultured in 6-well plates (Kasvi) with a cell density of approximately 5 × 10^5^ cells/cm^2^. After 24 h of treatments, total proteins were cold-extracted (with ice immersion) using a buffer containing 4 M urea, 2% SDS, 2 mM EGTA, 62.5 mM Tris-HCl pH 6.8, 2 mM EDTA, and 0.5% Triton X-100 and supplemented with 1 μL/mL of a protease inhibitor cocktail (Sigma-Aldrich, P8340). The experiments were performed in triplicate. The concentration of total proteins in the extracts obtained was quantified using the Lowry method. For Western blot analyses, proteins were separated by polyacrylamide gel electrophoresis and sodium dodecyl sulfate (SDS-PAGE) and electrotransferred to polyvinylidene difluoride (PVDF) membranes (Bio-Rad; Hercules, CA, USA). For immunodetection, the membranes were initially blocked in a buffer composed of 5% skim milk (Molico) in Tris-buffered saline with Tween 20 (TBS-T), containing 50 mM Tris-HCl, 150 mM NaCl, 0.05% Tween 20, and pH 7.4 (HCl) at 25 °C for 1 h. They were then incubated overnight at 4 °C with primary antibodies for STAT-3 (1:1000, Santa Cruz) and GAPDH (1:10,000, MERCK). The membranes were then washed three times with TBS-T and incubated for 1 h at room temperature with a secondary antibody anti-rabbit conjugated with peroxidase (1:5000; Molecular Probes, G21234) diluted in 5% skim milk TBS-T. After three washes with TBS-T and one wash with TBS, the membranes were incubated with the chemiluminescent substrate solution (ECL Plus Biorad Substrate Kit) for 5 min. Immunoreactive bands were then analyzed using the ImageQuant LAS 500 apparatus (GE Healthcare Life Sciences, Marlborough, MA, USA). The relative expression value of proteins was normalized according to the expression of GAPDH in the same sample. Quantification was obtained by densitometric scanning (ScanJet 4C, Hewlett Packard, Palo Alto, CA, USA) of three experiments and analyzed with ImageJ 1.33u software (Wayne Rasband, National Institutes of Health, Bethesda, MD, USA).

### 4.8. Statistical Analysis of Results

Data were statistically analyzed using GraphPad Prism 8 software (GraphPad, San Diego, CA, USA) for Windows. Experimental results are presented as means ± standard deviation (SD). To determine the statistical difference between the groups, analysis of variance was performed using a one-way ANOVA test, followed by Tukey’s post-hoc test for multiple comparisons. Parametric statistical tests were employed for comparisons between treatment groups and control groups. Statistical differences were considered significant at *p* ≤ 0.05. All experiments were repeated at least three times.

## 5. Conclusions

The results herein presented reinforce the anti-glioma potential of the flavonoid rutin and reveal its ability to modulate STAT3 signaling and the expression of onco miRNA-125b, which, through indirect interaction studies with microglia, is likely to impact the inflammatory profile of these cells towards a more antitumoral responsive phenotype. Rutin induced changes in the morphology of microglia in response to GBM cell treatment. Furthermore, the positive regulation of inflammatory mediators in microglia suggests a crucial role of rutin in modulating the local immune response. By negatively regulating the pro-tumorigenic signaling pathway STAT3 in microglia, rutin may have significant implications in suppressing tumor progression.

These findings provide valuable insights for the development of targeted therapies against GBM, which is known for its resistance to conventional treatments. The discoveries presented in this research indicate that rutin possesses properties capable of affecting multiple aspects of interactions between GBM cells and microglia, making it a promising substance for future investigations and the development of innovative therapeutic approaches.

## Data Availability

Data are contained within the article.

## References

[1] Wang H., Xu T., Huang Q., Jin W., Chen J. (2020). Immunotherapy for Malignant Glioma: Current Status and Future Directions. Trends Pharmacol. Sci..

[2] Grochans S., Cybulska A.M., Simińska D., Korbecki J., Kojder K., Chlubek D., Baranowska-Bosiacka I. (2022). Epidemiology of Glioblastoma Multiforme–Literature Review. Cancers.

[3] Virtuoso A., Giovannoni R., De Luca C., Gargano F., Cerasuolo M., Maggio N., Lavitrano M., Papa M. (2021). The Glioblastoma Microenvironment: Morphology, Metabolism, and Molecular Signature of Glial Dynamics to Discover Metabolic Rewiring Sequence. Int. J. Mol. Sci..

[4] Amici S.A., Dong J., Guerau-de-Arellano M. (2017). Molecular Mechanisms Modulating the Phenotype of Macrophages and Microglia. Front. Immunol..

[5] Rolle C.E., Sengupta S., Lesniak M.S. (2012). Mechanisms of Immune Evasion by Gliomas. Adv. Exp. Med. Biol..

[6] Solinas G., Germano G., Mantovani A., Allavena P. (2009). Tumor-Associated Macrophages (TAM) as Major Players of the Cancer-Related Inflammation. J. Leukoc. Biol..

[7] Wei J., Gabrusiewicz K., Heimberger A. (2013). The Controversial Role of Microglia in Malignant Gliomas. Clin. Dev. Immunol..

[8] Chang N., Ahn S.H., Kong D.-S., Lee H.W., Nam D.-H. (2017). The Role of STAT3 in Glioblastoma Progression through Dual Influences on Tumor Cells and the Immune Microenvironment. Mol. Cell Endocrinol..

[9] Kim J., Patel M., Ruzevick J., Jackson C., Lim M. (2014). STAT3 Activation in Glioblastoma: Biochemical and Therapeutic Implications. Cancers.

[10] Wu A., Wei J., Kong L.-Y., Wang Y., Priebe W., Qiao W., Sawaya R., Heimberger A.B. (2010). Glioma Cancer Stem Cells Induce Immunosuppressive Macrophages/Microglia. Neuro Oncol..

[11] Uddin M.S., Al Mamun A., Alghamdi B.S., Tewari D., Jeandet P., Sarwar M.S., Ashraf G.M. (2022). Epigenetics of Glioblastoma Multiforme: From Molecular Mechanisms to Therapeutic Approaches. Semin. Cancer Biol..

[12] Shi J. (2015). Regulatory Networks between Neurotrophins and MiRNAs in Brain Diseases and Cancers. Acta Pharmacol. Sin..

[13] Bartel D.P. (2004). MicroRNAs. Cell.

[14] Cao Q., Li Y.-Y., He W.-F., Zhang Z.-Z., Zhou Q., Liu X., Shen Y., Huang T.-T. (2013). Interplay between MicroRNAs and the STAT3 Signaling Pathway in Human Cancers. Physiol. Genom..

[15] Buruiană A., Florian Ș.I., Florian A.I., Timiș T.-L., Mihu C.M., Miclăuș M., Oșan S., Hrapșa I., Cataniciu R.C., Farcaș M. (2020). The Roles of MiRNA in Glioblastoma Tumor Cell Communication: Diplomatic and Aggressive Negotiations. Int. J. Mol. Sci..

[16] Gentile M.T., Ciniglia C., Reccia M.G., Volpicelli F., Gatti M., Thellung S., Florio T., Melone M.A.B., Colucci-D’Amato L., Ruta Graveolens L. (2015). Induces Death of Glioblastoma Cells and Neural Progenitors, but Not of Neurons, via ERK 1/2 and AKT Activation. PLoS ONE.

[17] Ganeshpurkar A., Saluja A.K. (2017). The Pharmacological Potential of Rutin. Saudi Pharm. J..

[18] Nasrabadi N.P., Zareian S., Nayeri Z., Salmanipour R., Parsafar S., Gharib E., Aghdaei A.H., Zali M.R. (2019). A Detailed Image of Rutin Underlying Intracellular Signaling Pathways in Human SW480 Colorectal Cancer Cells Based on MiRNAs-lncRNAs-mRNAs-TFs Interactions. J. Cell Physiol..

[19] Imani A., Maleki N., Bohlouli S., Kouhsoltani M., Sharifi S., Maleki Dizaj S. (2021). Molecular Mechanisms of Anticancer Effect of Rutin. Phytother. Res..

[20] Zhang P., Sun S., Li N., Ho A.S.W., Kiang K.M.Y., Zhang X., Cheng Y.S., Poon M.W., Lee D., Pu J.K.S. (2017). Rutin Increases the Cytotoxicity of Temozolomide in Glioblastoma via Autophagy Inhibition. J. Neurooncol.

[21] Lang G.-P., Li C., Han Y.-Y. (2021). Rutin Pretreatment Promotes Microglial M1 to M2 Phenotype Polarization. Neural Regen. Res..

[22] Santos B.L., Oliveira M.N., Coelho P.L.C., Pitanga B.P.S., da Silva A.B., Adelita T., Silva V.D.A., Costa M.d.F.D., El-Bachá R.S., Tardy M. (2015). Flavonoids Suppress Human Glioblastoma Cell Growth by Inhibiting Cell Metabolism, Migration, and by Regulating Extracellular Matrix Proteins and Metalloproteinases Expression. Chem. Biol. Interact..

[23] da Silva B.A., Coelho C.P.L., Amparo A.O.J., de Almeida A.M.M.C., Borges P.J.M., dos Santos C.S., Costa D.M.d.F., Mecha M., Rodriguez G.C., da Silva A.V.D. (2017). The Flavonoid Rutin Modulates Microglial/Macrophage Activation to a CD150/CD206 M2 Phenotype. Chem. Biol. Interact..

[24] de Amorim V.C.M., Júnior M.S.O., da Silva A.B., David J.M., David J.P.L., de Fátima M.D.C., Butt A.M., da Silva V.D.A., Costa S.L. (2020). Agathisflavone Modulates Astrocytic Responses and Increases the Population of Neurons in an in Vitro Model of Traumatic Brain Injury. Naunyn Schmiedebergs Arch. Pharmacol..

[25] da Silva A.B., Coelho C.P.L., das Neves M.O., Oliveira J.L., Oliveira J.A.A., da Silva K.C., Soares J.R.P., Pitanga B.P.S., dos Santos C.S., de Faria G.P.L. (2020). The Flavonoid Rutin and Its Aglycone Quercetin Modulate the Microglia Inflammatory Profile Improving Antiglioma Activity. Brain Behav. Immun..

[26] Santos B.L., Silva A.R., Pitanga B.P.S., Sousa C.S., Grangeiro M.S., Fragomeni B.O., Coelho P.L.C., Oliveira M.N., Menezes-Filho N.J., Costa M.F.D. (2011). Antiproliferative, Proapoptotic and Morphogenic Effects of the Flavonoid Rutin on Human Glioblastoma Cells. Food Chem..

[27] Xia H.-F., He T.-Z., Liu C.-M., Cui Y., Song P.-P., Jin X.-H., Ma X. (2009). *MiR-125b* Expression Affects the Proliferation and Apoptosis of Human Glioma Cells by Targeting *Bmf*. Cell. Physiol. Biochem..

[28] Smits M., Wurdinger T., Hof B., Drexhage J.A.R., Geerts D., Wesseling P., Noske D.P., Vandertop W.P., Vries H.E., Reijerkerk A. (2012). Myc-associated Zinc Finger Protein (MAZ) Is Regulated by MiR-125b and Mediates VEGF-induced Angiogenesis in Glioblastoma. FASEB J..

[29] Shi L. (2011). MicroRNA-125b-2 Confers Human Glioblastoma Stem Cells Resistance to Temozolomide through the Mitochondrial Pathway of Apoptosis. Int. J. Oncol..

[30] McFarland B.C., Hong S.W., Rajbhandari R., Twitty G.B., Gray G.K., Yu H., Benveniste E.N., Nozell S.E. (2013). NF-ΚB-Induced IL-6 Ensures STAT3 Activation and Tumor Aggressiveness in Glioblastoma. PLoS ONE.

[31] Parisi C., Napoli G., Pelegrin P., Volonté C. (2016). M1 and M2 Functional Imprinting of Primary Microglia: Role of P2X7 Activation and MiR-125b. Mediat. Inflamm..

[32] Kai K., Komohara Y., Esumi S., Fujiwara Y., Yamamoto T., Uekawa K., Ohta K., Takezaki T., Kuroda J., Shinojima N. (2022). Macrophage/Microglia-Derived IL-1β Induces Glioblastoma Growth via the STAT3/NF-ΚB Pathway. Hum. Cell.

[33] Sun Y., Liu W.-Z., Liu T., Feng X., Yang N., Zhou H.-F. (2015). Signaling Pathway of MAPK/ERK in Cell Proliferation, Differentiation, Migration, Senescence and Apoptosis. J. Recept. Signal Transduct..

[34] Nascimento R.P., Santos B.L., Silva K.C., Amaral da Silva V.D., Fátima Costa M., David J.M., David J.P., Moura-Neto V., Oliveira M.d.N., Ulrich H. (2021). Reverted Effect of Mesenchymal Stem Cells in Glioblastoma Treated with Agathisflavone and Its Selective Antitumoral Effect on Cell Viability, Migration, and Differentiation via STAT3. J. Cell Physiol..

[35] Rong X., Xu J., Jiang Y., Li F., Chen Y., Dou Q.P., Li D. (2021). Citrus Peel Flavonoid Nobiletin Alleviates Lipopolysaccharide-Induced Inflammation by Activating IL-6/STAT3/FOXO3a-Mediated Autophagy. Food Funct..

[36] Andersen R.S., Anand A., Harwood D.S.L., Kristensen B.W. (2021). Tumor-Associated Microglia and Macrophages in the Glioblastoma Microenvironment and Their Implications for Therapy. Cancers.

[37] Beurel E., Jope R.S. (2009). Lipopolysaccharide-Induced Interleukin-6 Production Is Controlled by Glycogen Synthase Kinase-3 and STAT3 in the Brain. J. Neuroinflam..

[38] Bocchini V., Casalone R., Collini P., Rebel G., Curto F. (1991). Lo Changes in Glial Fibrillary Acidic Protein and Karyotype during Culturing of Two Cell Lines Established from Human Glioblastoma Multiforme. Cell Tissue Res..

[39] Garcia-Mesa Y., Jay T.R., Checkley M.A., Luttge B., Dobrowolski C., Valadkhan S., Landreth G.E., Karn J., Alvarez-Carbonell D. (2017). Immortalization of Primary Microglia: A New Platform to Study HIV Regulation in the Central Nervous System. J. Neurovirol..

[40] Schmittgen T.D., Livak K.J. (2008). Analyzing real-time PCR data by the comparative CT method. Nat. Protoc..

